# Factors Associated With Deep Sedation Practice in Mechanically Ventilated Patients: A *Post hoc* Analysis of a Cross–Sectional Survey Combined With a Questionnaire for Physicians on Sedation Practices

**DOI:** 10.3389/fmed.2022.839637

**Published:** 2022-06-09

**Authors:** Penglin Ma, Tao Wang, Yichun Gong, Jingtao Liu, Wei Shi, Lin Zeng

**Affiliations:** ^1^Critical Care Medicine Department, Guiqian International General Hospital, Guiyang, China; ^2^Surgical Intensive Care Unit (SICU), The 8th Medical Center of General Hospital of Chinese People's Liberation Army, Beijing, China; ^3^Research Center of Clinical Epidemiology, Peking University Third Hospital, Beijing, China

**Keywords:** deep sedation practice, patient tolerability, stressful stimuli, physician's perception, mechanical ventilation

## Abstract

**Purpose:**

The study aimed to explore factors associated with deep sedation practice in intensive care units (ICUs).

**Materials and Methods:**

A *post hoc* analysis was conducted for a cross–sectional survey on sedation practices in mechanically ventilated (MV) patients, combined with a questionnaire for physicians regarding their preferences for light sedation (P–pls Score) in 92 Chinese ICUs.

**Results:**

There were 457 and 127 eligible MV patients in the light and deep sedation groups respectively. A multivariable logistic regression analysis demonstrated that the control mode of mechanical ventilation, plasma lactate level, and the Sequential Organ Failure Assessment (SOFA) score were independent risk factors for deep sedation practice (*p* <0.01). Notably, the adjusted odds ratio (95% CI) of the average P–pls score in the ICU ≤ 2 for deep sedation practice was 1.861 (1.163, 2.978, *p* = 0.01). In addition, the areas under curves of receiver operating characteristics (AUC–ROC) of the model to predict the probability of deep sedation practice were 0.753 (0.699, 0.806) and 0.772 (0.64, 0.905) in the training set and the validation set, respectively. The 28–day mortality was increased in patients with exposure to deep sedation practice but not significantly.

**Conclusion:**

Both factors related to stressful stimuli and the ICU physicians' perception of patient tolerability in mechanical ventilation were likely associated with deep sedation practice in MV patients.

## Introduction

It was previously documented that mechanically ventilated (MV) and critically ill patients were deeply sedated (defined as the Richmond Agitation–Sedation Scale equal to or < −3, RASS ≤ −3) very frequently (1–3). Compared with light levels of sedation (i.e., RASS ranged from −2 to 1, largely), significantly, deep sedation has been associated with poor outcomes including prolonged duration of mechanical ventilation, increased incidence of ventilator–associated pneumonia (VAP), declined cognitive ability, and even increased long–term mortality (1–5). Moreover, it was recently demonstrated that the implementation of no sedation protocol resulted in more days free from coma or delirium than the light sedation strategy during the stay in the ICUs (6). Therefore, deep sedation is mostly unnecessary and should be avoided by the implementation of a minimal sedation strategy, including light sedation protocol and the early Comfort using Analgesia, minimal Sedatives, and maximal Humane care (eCASH) concept in ICU MV patients (7–9).

However, at present, the frequency of deep sedation remains high in clinical practice. Fuller et al. reported that the prevalence of deep sedation was 52.8% (171/324) in a consecutive cohort of MV patients in the emergency department (ED) (10). Significantly, deep sedation was continued in 75% of the patients (92/171) on ICU day 1 in this cohort. Moreover, the depth of sedation was determined as RASS < −2 (mean RASS = −2.3) on day 1 in the light sedation group of Olsen's randomized control trial (RCT) on no sedation or light sedation in critically ill and MV patients (6). Low adherence to the minimal sedation strategy was previously attributable to inadequate assessments due to a shortage of nurses, lack of multidisciplinary cooperation, and even misperception (11–14). However, there is a paucity of research to comprehensively interpret the fact that care providers deepen sedation at RASS < −2 for MV patients frequently. It was proposed that several factors, including the severity of pathophysiological alternations, the intensity of supportive therapies, and ICU physicians' perception of patients' tolerability to light sedation, were involved in the care providers' decision–making for sedation depth in MV patients. Therefore, as an extension of the previous study, we did a *post hoc* analysis of a nationwide cross–sectional study combined with a questionnaire survey to investigate the factors associated with deep sedation practice in MV patients.

## Methods

### Study Design and Setting

The *post hoc* analysis, which included a 24–h survey on real sedation practices in MV patients and a questionnaire for physicians regarding their preferences for light sedation, was conducted on 92 Chinese ICUs on 11 May 2016. Ethical committee approval was obtained from each participating hospital. Informed consent was waived by the ethics committees of all the participating hospitals because of the observational nature of this study. A site investigator was responsible for this study in each recruited ICU. Additionally, a clinical research coordinator (CRC) was assigned to each ICU to ensure the quality of data collection and to perform the questionnaire survey simultaneously. This study was registered on the website of www.chictr.org.cn (registration number: ChiCTR–EOC−16008444).

### Patient Recruitment, Data Collection, and Questionnaire Survey

**Figure 1:**
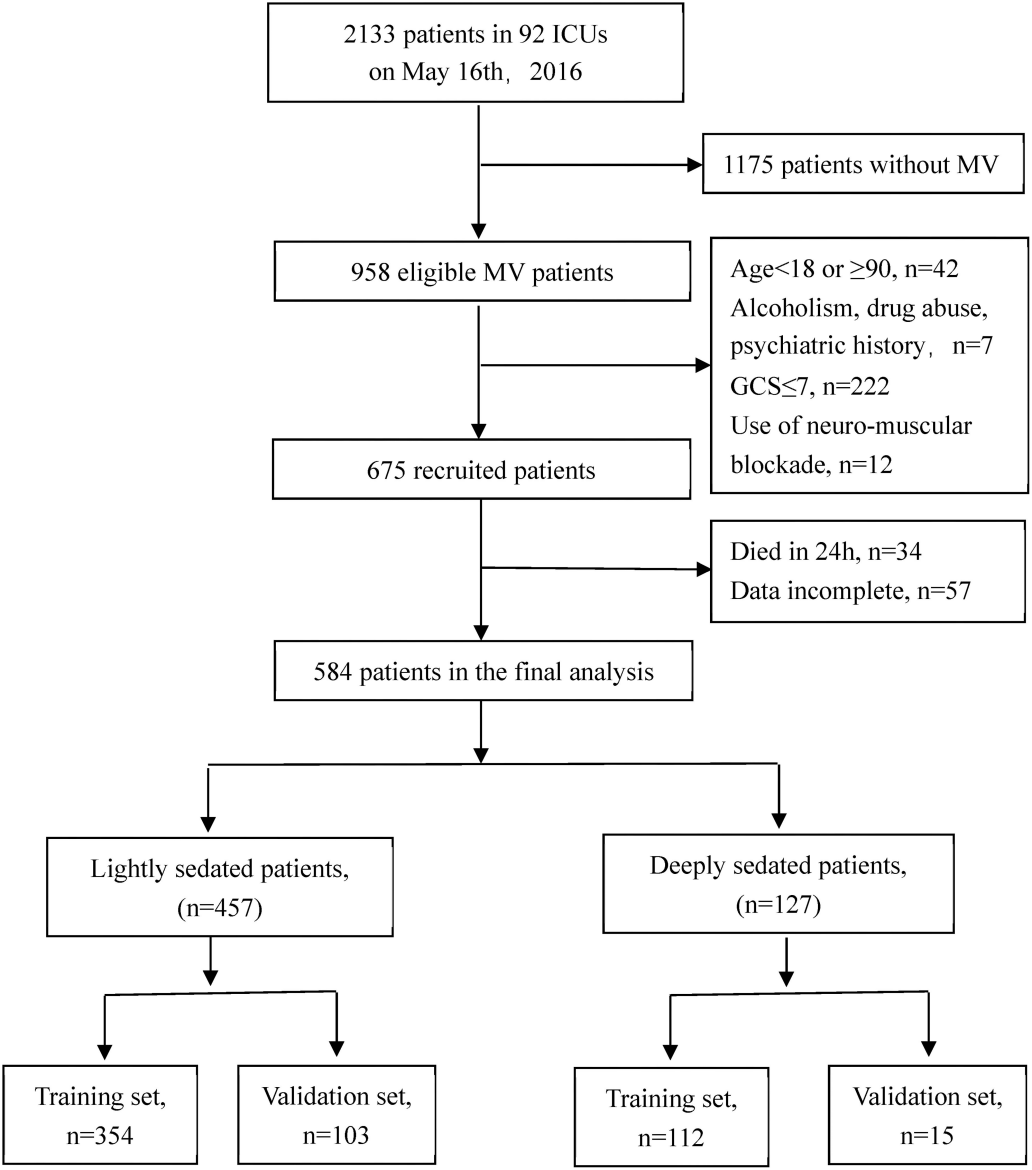
Flow diagram of the study.

All patients on invasive mechanical ventilation were eligible to be enrolled in this study. The exclusion criteria were people aged younger than 18 or over 90 years, those with a Glasgow Coma Scale (GCS) score ≤ 7, and people with history of alcoholism, drug abuse, psychiatric illness, severe acute respiratory distress syndrome (ARDS), or use of neuromuscular blockade (Figure 1). Patients who died within 24 h were eliminated. Intensive care was provided as usual for all the recruited patients in each participating center.

The observation was initiated at 6:00 AM on the study day until 5:00 AM the next day. Demographics and characteristics of the patients were collected. Three measurements of RASS were required for each enrolled patient, at 6:00–7:00, 13:00–14:00, and 22:00–23:00, no matter how frequently RASS was assessed in usual care. Body temperature, ventilator settings, and the Sequential Organ Failure Assessment (SOFA) score were repeatedly evaluated while assessing RASS. The Acute Physiology and Chronic Health Evaluation II (APACHE II) score within 24 h of ICU admission was recorded. Agitation was recorded within this 24–h observational period. The confusion assessment method for the intensive care unit (CAM–ICU) was used to evaluate delirium while the patients were agitated. Pain assessment was reviewed by the CRC the next day.

Patients recorded with all required RASS assessments ≥ −2 composed the light sedation group; otherwise, they were classified into the deep sedation group (i.e., patients with one record of RASS < −2 at least).

Up to 6 physicians in each recruited ICU were surveyed simultaneously. The questionnaire was developed based on the results of Delphi processing in a panel of experts and testing on 63 doctors. ICU physicians' preference for light sedation (P–pls score) was calculated by selected answers for 10 specific items of this questionnaire, which was described in detail in our previous study (15). The average P–pls score of each recruited ICU was calculated by the sum of P–pls score divided by the number of physicians completing this survey in the ICU in this *post hoc* analysis.

### Statistical Analysis

The number of events per variable (EPV) in logistic regression analysis was used to estimate the sample size of this study (16). According to our previous publications (15, 17), there were no more than 12 variables that would be considered to potentially impact decision–making for the depth of sedation and included in the logistic regression model. Accordingly, at least 120 events (patients with deep sedation) should be collected. With a deep sedation rate of about 23%, a total number of 522 eligible patients should be enrolled in this study.

In fact, 80% of the patients were randomly selected as the training set and twenty percent as the validation set. Patients' characteristics, such as age, gender, highest body temperature, and disease type, were described according to data distributions. Continuous variables that followed a Gaussian distribution were described as means and standard deviation and compared by Student's *t*–test. When continuous variables were not normally distributed, they were expressed as median (minimum, maximum) and were compared using rank–sum tests. Categorical variables were described as numbers and proportions and were compared using the Chi–square test or Fisher's exact test as appropriate.

Based on the training set, we tried to establish a multivariable logistic regression model to confirm the associate factors that impact the decision–making of deep sedation. Variables with *p*–values <0.1 and factors that were considered as a potential impact factor in previous studies, such as highest body temperature, were included in the logistic regression model. For this purpose, logistic regression with backward selection was conducted. The receiver operating characteristics (ROC) curve of the training set and the verification set were drawn, and areas under the ROC (AUC–ROC) curves were calculated to assess the accuracy of the prediction model. All statistical analyses were performed in SPSS v. 25.0. A two–sided *p*–value <0.05 was regarded as statistically significant.

## Results

### Patients

A total of 2,133 patients were screened in this study. After applying the inclusion and exclusion criteria, 584 eligible MV patients from 92 ICUs were finally enrolled, including 457 in the light sedation group and 127 in the deep sedation group (Figure 1). Baseline comparisons between the light and deep sedation groups in either the training set or the validation set are shown in Table 1. The distribution of gender, category of diseases, and age was similar between the light and deep sedation groups. Characteristics of patients such as with/without pain assessment, highest body temperature, positive end–expiratory pressure (PEEP) level, plateau pressure (Plat–p), PaO_2_, respiratory rate (RR), and minute ventilation (Min–vent) within the observation period, APACHE II score, and average P–pls score in the recruited ICU were not found to be significantly different in the deep sedation group compared with those in the light sedation group (*p* >0.05, Table 1). Meanwhile, there were significant differences in the proportion of patients with septic shock and those receiving the control mode (referenced to the assisted mode) of mechanical ventilation, SOFA score, level of variables related to oxygenation [FiO_2_ and P/F ratio (PaO_2_/FiO_2_)], and circulatory function (norepinephrine dosage and plasma lactate level) between the light and deep sedation groups in the training set (Table 1). Additionally, the proportion of patients recruited from the ICUs characterized with an average of P–pls score ≥ 2 was significantly higher in the light sedation group than in the deep sedation group (62.4% vs. 46.4%, *p* = 0.005) in the training set.

**Table 1:** Baseline characteristics of mechanically ventilated (MV) patients in the training set and the validation set.

	**Training set**			**Validation set**		
	**Light sedation**	**Deep sedation**		**Light sedation**	**Deep sedation**
	**(*n* = 354)**	**(*n* = 112)**	**P**	**(*n* = 103)**	**(*n* = 15)**	**P**
**High–T (C°)**, Mean (SD)	36.9 (0.9)	37.0 (0.9)	0.474	37.0 (0. 9)	36.7 (0.8)	0.192
**Gender**, n(%)						
Male	243 (68.6%)	74 (66.1%)	0.611	64 (62.1%)	7 (46.7%)	0.253
Female	111 (32.0%)	38 (33.9%)		39 (37.9%)	8 (53.3%)	
**Age** Mean (SD)	61.7 (17.7)	61.5 (15.6)	0.920	63.7 (17.5)	67.2 (15.5)	0.462
**Category of disease**, *n*(%)						
Surgical	200 (56.5%)	64 (57.1%)	0.904	51 (49.5%)	5 (33.3%)	0.241
Medical	154 (43.5%)	48 (42.9%)		52 (50.5%)	10 (66.7%)	
**Septic shock**, *n*(%)	40 (11.3%)	22 (19.6%)	0.023	9 (8.7%)	5 (33.3%)	0.006
**SOFA**, Median (range)	4.0 (0.0, 18.0)	7.0 (0.0, 18.0)	<0.001	4.0 (0.0, 17.0)	8.0 (2.0, 15.0)	<0.001
Mean (SD)	5.0(3.2)	7.2(3.6)	<0.001	4.8(3.2)	8.1(3.2)	<0.001
**APACHE II**, Median (range)	14.0 (1.0, 37.0)	15.5 (4.0, 33.0)	0.351	15.0 (3.0, 39.0)	22.0 (8.0, 33.0)	0.008
Mean (SD)	15.3(7.4)	16.0(7.1)	0.405	16.2(7.3)	22.1(7.9)	0.005
**Mode of MV**, *n*(%)						
Assisted	285 (80.5%)	59 (52.7%)	<0.001	83 (80.6%)	7 (46.7%)	0.004
Control	69 (19.5%)	53 (47.3%)		20 (19.4%)	8 (53.3%)	
**PEEP** (cmH_2_O), Median (range)	5.0 (0.0, 20.0)	5.0 (0.0, 14.0)	0.081	5.0 (0.0, 14.0)	5.00 (2.0, 10.0)	0.363
Mean (SD)	5.3(2.2)	5.8(2.6)	0.050	4.9(2.2)	5.5(2.2)	0.312
**Plat–p** (cmH_2_O), Median (range)	17.0 (9.0, 36.0)	17.0 (7.1, 38.0)	0.774	16.0 (7.0, 33.0)	20.0 (10.0, 32.0)	0.047
Mean (SD)	17.8(5.1)	18.3(6.6)	0.415	17.0(5.0)	20.2(6.7)	0.027
**PaO**_**2**_(mmHg), Median (range)	101.0 (52.6, 410.0)	96.5 (46.0, 240.0)	0.165	100.0 (54.0, 267.0)	86.9 (62.5, 169.0)	0.039
Mean (SD)	109.4(36.0)	105.3(36.4)	0.300	111.4(38.6)	92.9(27.1)	0.076
**FiO**_**2**_ (%), Mean (SD)	43.5 (10.9)	49.1 (15.3)	<0.001	43.5 (9.9)	43.7 (7.4)	0.955
Mean (SD)	43.5(10.9)	49.1(15.3)	<0.001	43.5(9.9)	43.7(7.4)	0.963
**P/F ratio,**Median (range)	250.0 (59.2, 487.5)	210.6 (68.8, 495.0)	<0.001	245.0 (106.5, 473.3)	211.5 (125.0, 338.0)	0.039
Mean (SD)	260.4(83.8)	230.5(95.3)	0.002	259.9(80.7)	216.8(63.6)	0.050
**RR** (breaths/min), Median (range)	17.0 (6.0, 41.0)	16.0 (11.0, 35.0)	0.118	18.0 (11.0, 37.0)	18.0 (13.0, 34.0)	0.694
Mean (SD)	18.1(5.3)	17.7(5.5)	0.453	18.7(5.0)	20.1(6.8)	0.328
**Min–vent** (L/min), Median (range)	8.00 (4.30, 19.20)	7.90 (4.80, 17.60)	0.881	8.20 (4.30, 18.90)	8.69 (4.93, 16.70)	0.340
Mean (SD)	8.40(2.24)	8.55(2.61)	0.583	8.71(2.61)	10.04(3.99)	0.227
**NE dosage**,Median (range)	0.0 (0.0, 0.7)	0.0 (0.0, 3.0)	0.001	0.0 (0.0, 2.0)	0.0 (0.0, 0.5)	0.025
Mean (SD)	0.04(0.10)	0.10(0.30)	0.070	0.06(0.23)	0.13(0.17)	0.307
**Lac (mmol/L)**, Median (range)	1.3 (0.3, 10.8)	1.9 (0.5, 15.8)	<0.001	1.5 (0.3, 8.6)	1.2 (0.7, 6.4)	0.622
Mean (SD)	1.6(1.2)	2.8(2.9)	<0.001	1.6(1.1)	1.9(1.5)	0.451
**RASS**, Median (range)	0.0 (−2.0, 4.0)	−3.0 (−5.0, −3.0)	<0.001	0.0 (−2.0, 4.0)	−3.0 (−5.0, −3.0)	<0.001
Mean (SD)	−0.4(1.2)	−3.4(0.6)	<0.001	−0.5(1.3)	−3.3(0.62)	<0.001
**Agitation**, n(%)	131 (37.0%)	11 (9.8%)	<0.001	37 (35.9%)	1 (6.7%)	0.023
**Pain assessment**, n(%)	112 (31.6%)	40 (35.7%)	0.423	29 (28.2%)	9 (60.0%)	0.014
**P–pls score**, Median (range)	3.1 (−5.0, 7.0)	2.0 (−2.0, 7.0)	0.193	2.8 (−4.0, 6.5)	3.2 (−2.0, 5.8)	0.598
Mean (SD)	2.6(2.6)	2.3(2.5)	0.398	2.7(2.5)	3.0(2.3)	0.633
>2, n(%)	221 (62.4%)	52 (46.4%)	0.003	69 (67.0%)	10 (66.7%)	0.980
≤ 2, n(%)	133 (37.6%)	60 (53.6%)		(33.0%)	5 (33.3%)	

*High–T means highest body temperature within the observation period; SOFA, Sequential Organ Failure Assessment score; APACHE II, Acute Physiology And Chronical Health Evaluation II score; Plat–p, plateau pressure; P/F ratio was calculated by PaO_2_ divided by FiO_2_; RR, respiratory rate; MV, mechanical ventilation; Min–vent, minute ventilation; NE, norepinephrine; PEEP, positive end–expiratory pressure; M (range), median (minimal, maximal); P–pls score was the average score of P–pls (physician's preference to light sedation) in the ICU where the patient was admitted*.

### Outcomes Associated With Sedation Depth

The 28–day mortality and the prevalence of delirium during ICU stay are listed in Table 2. The 28–day mortality was significantly lower in the light sedation group than in the deep sedation group [10.7% vs. 19.7%, crude OR = 2.218 (1.251, 3.62)]. However, the prevalence of delirium within the observation day increased in the light sedation group in comparison with the deep sedation group [4.2% vs. 0.8%, crude OR (95% CI) = 0.269 (0.035, 2.046)]. By multivariable logistic regression, the adjusted OR (95% CI) of deep sedation for 28–day mortality and delirium was 1.492 (0.828, 2.688) and 0.273 (0.031, 2.382), respectively. Differences between the light sedation group and the deep sedation group were not statistically significant.

**Table 2:** Risk potential of deep sedation for outcomes in MV patients(*n* = 584).

	**Light sedation**	**Deep sedation**	**Crude OR (95% CI)**	**Adjusted OR (95% CI)**
**Delirium**	19/457 (4.2%)	1 /127(0.8%)	0.269 (0.035, 2.046)	0.273 (0.031, 2.382)
**28–day death**	49/457 (10.7%)	25/127 (19.7%)	2.128 (1.251, 3.620)	1.492(0.828, 2.688)

*Adjusted odds ratios (ORs) were adjusted for gender, age, category of disease, septic shock, SOFA (Sequential Organ Failure Assessment) score, APACHE II (Acute Physiology And Chronical Health Evaluation II) score, P/F ratio (calculated by PaO_2_ divided by FiO_2_), infused norepinephrine dosage, and plasma lactate level. Light sedation was used as the reference for estimation of OR*.

### Factors Associated With Deep Sedation Practice

The multivariable logistic regression analysis demonstrated that the control mode of mechanical ventilation, plasma lactate level, and SOFA score were independent risk factors associated with deep sedation practice (*p* < = 0.01, Table 3). In addition, the adjusted odds ratio (95% CI) of the average ICU P–pls score ≤ 2 for deep sedation practice was 1.861 (1.163, 2.978, *p* = 0.01).

**Table 3:** Independent risk factors for deep sedation practice (training set, *n* = 466).

	**β**	**Odds Ratio (95% CI)**	**p**
**P–pls (≤2 vs**. **>2)**	0.621	1.861 [1.163,2.978]	0.010
**Control Mode of mechanical ventilation**	0.958	2.608 [1.591,4.275]	<0.001
**Lactate(mmol/L)**	0.245	1.278 [1.108,1.472]	0.001
**SOFA**	0.139	1.149 [1.071,1.231]	<0.001

*Multivariable logistic regression was conducted for analyzing independent risk factors for deep sedation practices in the training set. P–pls score was the average score of P–pls (physician's preference for light sedation) in the ICU where the patient was admitted. SOFA, Sequential Organ Failure Assessment score*.

**Figure 2:**
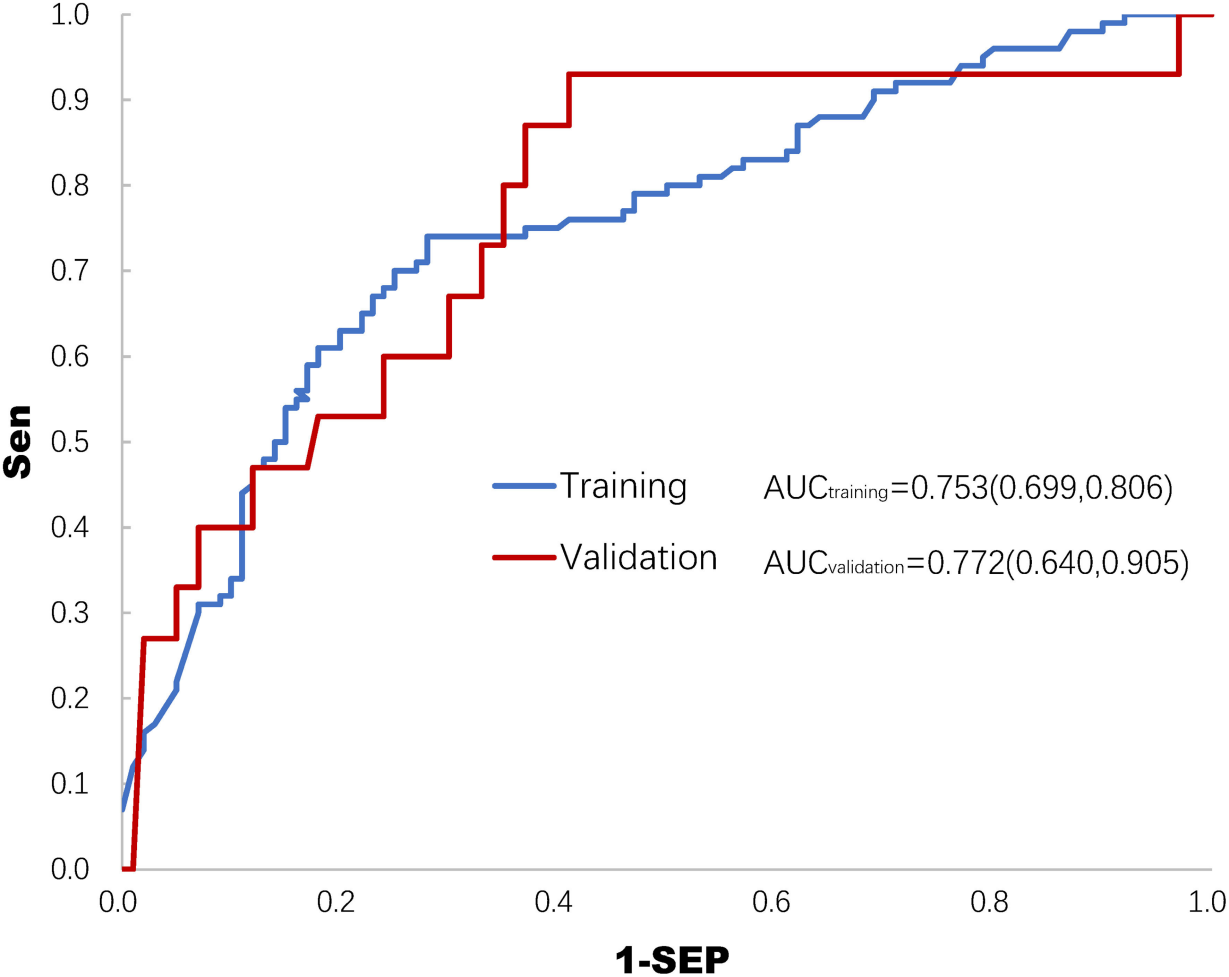
Receiver operating characteristics (ROC) curve of the training set and the validation set for predicting deep sedation practice. AUC, area under the curve of ROC.

After establishing the prediction model of the training set, prediction probabilities were estimated in the validation set. The ROC curves of both the training set and the validation set are shown in Figure 2. The AUCs of ROC were 0.753 (0.699, 0.806) and 0.772 (0.640, 0.905) in the training set and the validation set, respectively (Figure 2).

## Discussion

The main results of this study demonstrated that factors including the SOFA score, plasma lactate level, mode of mechanical ventilation, and average P–pls (physician's preference to light sedation) score in the ICU were significantly associated with deep sedation practice (usually defined as RASS < −2), which likely worsened the 28–day mortality in MV patients. In addition, a logistic regression model was developed and validated to predict the probability of deep sedation practice despite the AUC–ROC being 0.753 (0.699, 0.806) and 0.772 (0.640, 0.905) in the *post hoc* analysis (Figure 2). These findings provided important information that either the intensity of stressful stimuli or the ICU physicians' perception of patient tolerability in mechanical ventilation was involved in decision–making for deep sedation practice in MV patients.

The use of analgesics and sedatives is aimed at regulating patient discomfort (18), which is caused by stressful stimuli including both physiological stresses induced by pathophysiological abnormalities or/and intensive care and mental stress (18–21). Meanwhile, overuse of sedatives and analgesics is harmful to host defenses and leads to worse outcomes for MV patients (22, 23). Up to now, increasing data, including the results in this study, have revealed a direct relationship between sedation depth and clinical outcomes (24, 25). These results indicated that MV patients were largely at risk potential for overuse of sedatives and analgesics while RASS was scored below −2. Based on the opinions of experts, deep sedation was recommended only for managing a few specific situations in MV patients, such as severe acute respiratory distress syndrome (ARDS) with ventilator–patient asynchrony or use of neuromuscular blocking agents, severe brain injury with severe intracranial hypertension, and status epilepticus (26–29). Meanwhile, indications for deep sedation or contraindications for maintaining MV patients at light levels of sedation remain unexplained (30). One of the important barriers is how to define the intensity of stimuli–induced discomfort requiring deep levels of sedation. Although a variety of stressful stimuli was reported (20, 21, 31), tools to scale the intensity of stimuli are yet to be developed. In fact, few research studies previously provided evidence regarding the estimation of stressful stimuli and investigated the dose responses of either sedatives or analgesics (or the combined use of both drugs) against the stimuli. A strength of this study was that some stressful stimuli, in terms of high plasma lactate level, increase in SOFA score, and use of the control mode of mechanical ventilation (shown in Table 3), were demonstrated as independent risk factors for deep sedation practice in the management of MV patients. This finding suggested that the intensity of these stressful stimuli should be considered while care providers are titrating the depth of sedation for MV patients. Based on these variables, we recently developed an ensemble model for the prediction of agitation in MV patients who were sedated at light levels of sedation in the same cohort (17). The results indicated that the stressful stimuli could not be successfully attenuated by titrating sedatives as well as analgesics while the MV patients who were predicted with a high risk of agitation were lightly sedated (RASS ≥ 2). Therefore, an investigation on the relationship between the intensity of stressful stimuli and the levels of sedation depth is necessary for defining the stimuli–based indications of deep sedation in further study, which will help to promote sedation practices in MV patients.

In addition, it was demonstrated that the physicians' perception of patient tolerance to stressful stimuli was involved in the decision–making for deep sedation practice. In fact, assessment of patients' tolerability during mechanical ventilation remains problematic. By titrating analgesics and sedatives, in clinical practice, a calm and cooperative status was estimated as patient tolerable in mechanical ventilation. Among assessment tools, RASS, which offers broader discrimination in the mild–to–moderate sedation range, is the most commonly used and reliable one to evaluate patient tolerability in mechanical ventilation (32–34). However, the result of the RASS assessment is the transient tolerability of MV patients regulated by the infused analgesics and sedatives. While the intensity of nociceptive stimuli changes because of the occurrence or disappearance of fever, thirst, drainage tube pain, andintestinal colic as well as upregulation or downregulation of supportive therapies such as changes in ventilator settings, significantly, MV patients would become over—or under–sedated as the dosages of sedatives and analgesics were unchanged (30). This partially at least accounts for the frequent and unpredictable agitation as well as oversedation in the real practice. Lacking a reliable tool to scale patient tolerance to stressful stimuli instantly, care providers always face a big challenge in titrating sedatives and analgesics to regulate patient discomfort during mechanical ventilation. The decision–making for the depth of sedation was more likely dependent on their own recognitions and experiences. As reported in previous studies, care providers' concern about patient intolerance to mechanical ventilation such as agitation was an important barrier to the implementation of a minimal sedation strategy (15, 35). Therefore, the development of evidence–based deep sedation indications is critical to avoiding unjustified deep sedation practices in MV patients. As the basis for this task, it is necessary to investigate stressful stimuli, sedative choice (either type or dosage), and patient tolerability as well as their interactions in further research studies (36).

Some limitations to our study should be addressed. First, this is a *post hoc* analysis of a study aimed at developing a model for the prediction of agitation in MV patients maintained under light sedation (30). Some important measurements such as anxiety score and pain score were not collected in this study. In addition, the instant changes of variables before patients were deeply sedated, such as agitation and asynchrony, which would help to spot if the deep sedation was appropriate or not, were not systematically considered in the primary study protocol. Lack of data on these factors was the most important limitation of this study, accounting for partially, at least, the AUC–ROC of the logistic regression model being less powerful [0.753 (0.699, 0.806)] to predict deep sedation. However, the findings of this study demonstrated the feasibility of a prospective, multicentered, large–scale cohort study to define stimulus–based indications for deep sedation in further research. Second, several factors contributed to the low rate of delirium in this study, including excluding patients with delirium assessment reporting positive before the cross–sectional survey, delirium being assessed only within the 24–h observational period, and CAM–ICU assessment being problematic in the deeply sedated MV patients. These are the reasons for the low incidence of delirium in this cohort. Significantly, the lower delirium rate in the deep than in the light sedation group was largely caused by the failure in CAM-ICU assessment in most of the deeply sedated MV patients. Finally, the total dosage of sedatives and analgesics was not provided, which could be used as direct evidence of oversedation.

## Conclusion

The results of this study demonstrated that, in the 24–h survey, deep sedation practice was frequent and likely worsened 28–day mortality in MV patients. Factors related to the intensity of stressful stimuli such as severity of pathophysiological alternations and intensity of supportive therapies were significantly associated with the probability of deep sedation practice in MV patients. Additionally, the ICU physicians' perception of patients' tolerability in mechanical ventilation was involved in decision–making for deep sedation practice. Besides our findings in this study, meanwhile, several factors could contribute to the behavior of intensive care providers toward deepening sedation (the primary outcome) in clinical practice. These findings suggest that the development of evidence–based deep sedation indications is feasible and, notably, critical to avoid unjustified deep sedation practices in MV patients.

## Data Availability Statement

The raw data supporting the conclusions of this article are available from the corresponding author upon reasonable request.

## Ethics Statement

Our study involving human participants was reviewed and approved by the Ethical Committees of all participating hospitals (approval number from the principal center: 309201906171118). Informed consent was waived by the ethics committees. The list of 80 participating hospitals (recruiting 92 ICUs) was as follows:

1. The 8th Medical Center of General Hospital of Chinese People's Liberation Army, 100091, Beijing, P. R. China; 2. Peking University Third Hospital; 3. Peking University First Hospital; 4. Peking University People's Hospital; 5. Peking Union Medical College Hospital; 6. Fuwai Hospital Chinese Academy of Medical Sciences; 7. Beijing Tsinghua Changgung Hospital; 8. Beijing Anzhen Hospital of Capital Medical University; 9. Beijing Chaoyang Hospital of Capital Medical University; 10. Beijing TianTan Hospital of Capital Medical University; 11. Beijing Fuxing Hospital of Capital Medical University; 12. General Hospital of Chinese People's Liberation Army; 13. Beijing Youyi Hospital of Capital Medical University; 14. China-Japan friendship hospital; 15. The Fourth Clinical Hospital affiliated to Harbin Medical University; 16. The Third Clinical Hospital affiliated to Harbin Medical University; 17. The Second Clinical Hospital affiliated to Harbin Medical University; 18. The First Clinical Hospital affiliated to Harbin Medical University; 19. Bethune First Hospital Of Jilin University; 20. The Second Hospital of Jilin University 21. The First Affiliated Hospital of Dalian Medical University; 22. The First Affiliated Hospital of Liaoning Medical College; 23. General Hospital of the northern theater Military Region; 24. The First Hospital of China Medical University; 25. Shengjing Hospital of China Medical University; 26. First Hospital of China Medical University; 27. Hebei General Hospital; 28. Hebei Kailuan General Hospital; 29. The fourth hospital of Hebei Medical University; 30. The third hospital of Hebei Medical University; 31. Tianjin Third General Hospital; 32. Fujian Provincial Hospital; 33. The First Affiliated Hospital of Fujian Medical University; 34. The First Affiliated Hospital of Xiamen University; 35. Xiamen Cardiovascular Hospital of Xiamen University; 36. Shanghai Changzheng Hospital; 37. Zhongshan Hospital Fudan University; 38. Renji Hospital Affiliated to Shanghai Jiaotong University School of Medicine; 39. Ruijin Hospital Affiliated to Shanghai Jiaotong University School of Medicine; 40. Xinhua Hospital Affiliated to Shanghai Jiaotong University School of Medicine; 41. The First Affiliated Hospital of Zhejiang University; 42. The Second Affiliated Hospital of Zhejiang University; 43. Zhejiang Provincial People's Hospital; 44. The Institute of Gerontology of Guangdong Provincial People's Hospital; 45. General Hospital of Southern Treater Command; 46. The First Affiliated Hospital of Guangzhou Medical University; 47. The First Affiliated Hospital of Sun Yat-sen University; 48. The First Affiliated Hospital of Guangxi Medical University; 49. The Affiliated Hospital of Guizhou Medical University; 50. Hainan General Hospital; 51. Qingdao Municipal Hospital 52. Qilu Hospital of Shandong University; 53. Shandong Provincial Hospital; 54. Liaocheng People's Hospital; 55. Yantai Yuhuangding Hospital; 56. Zibo Central Hospital; 57. The First Affiliated Hospital of Bengbu Medical College; 58. Anhui Provincial Hospital; 59. Zhongda Hospital Southeast University; 60. General Hospital of Eastern Treater Command; 61. Suzhou Municipal Hospital; 62. Henan Provincial People's Hospital; 63. The First Affiliated Hospital of Zhengzhou University; 64. Xijing Hospital; 65. Wulumuqi General Hospital of Chinese PLA; 66. First Affiliated Hospital, School of medicine, Shihezi University; 67. The First Affiliated Hospital of Xingjiang Medical University; 68. Sichuan Provincial People's Hospital; 69. West China Hospital of Sichuan University; 70. The First Affiliated Hospital of Kunming Medical University; 71. Army Medical Center of Chinese PLA; 72. The First Affiliated Hospital of Chongqing Medical University; 73. Renmin Hospital of Wuhan University; 74. Zhongnan Hospital of Wuhan University; 75. Union Hospital, Tongji Medical College, Huazhong University of Science and Technology; 76. The Second Affiliated Hospital of South China University; 77. The Second Xiangya Hospital of Central South University; 78. The Third Xiangya Hospital of Central South University; 79. The Xiangya Hospital of Central South University; 80. The First Affiliated Hospital of Nanchang University.

## Author Contributions

PM, LZ, and WS were the major contributors in designing, conducting this study and writing the manuscript. TW, YG, and JL participated in conducting this study and helped to revise the manuscript. All authors read and approved the submitted manuscript.

## Funding

Capital's Funds for Health Improvement and Research, Grant No: [CFH2018–2–4098].

## Conflict of Interest

The authors declare that the research was conducted in the absence of any commercial or financial relationships that could be construed as a potential conflict of interest.

## Publisher's Note

All claims expressed in this article are solely those of the authors and do not necessarily represent those of their affiliated organizations, or those of the publisher, the editors and the reviewers. Any product that may be evaluated in this article, or claim that may be made by its manufacturer, is not guaranteed or endorsed by the publisher.
